# Comparison in Conscious Rabbits of the Baroreceptor-Heart Rate Reflex Effects of Chronic Treatment with Rilmenidine, Moxonidine, and Clonidine

**DOI:** 10.3389/fphys.2016.00522

**Published:** 2016-11-15

**Authors:** Monique L. Parkin, Kyungjoon Lim, Sandra L. Burke, Geoffrey A. Head

**Affiliations:** Neuropharmacology Laboratory, Baker IDI Heart and Diabetes InstituteMelbourne, VIC, Australia

**Keywords:** moxonidine, rilmenidine, clonidine, baroreceptor-heart rate reflex, blood pressure, conscious rabbits, cardiac vagus, cardiac sympathetic

## Abstract

We investigated the effects of chronic subcutaneous treatment with centrally-acting antihypertensive agents moxonidine, rilmenidine, and clonidine on the baroreflex control of heart rate (HR) in conscious normotensive rabbits over 3 weeks. Infusions of phenylephrine and nitroprusside were performed at week 0 and at weeks 1 and 3 of treatment to determine mean arterial pressure (MAP)-HR baroreflex relationships. A second curve was performed after intravenous methscopolamine to determine the sympathetic baroreflex relationship. The vagal component of the reflex was determined by subtracting the sympathetic curve from the intact curve. Clonidine and moxonidine (both 1 mg/kg/day), and rilmenidine (5 mg/kg/day), reduced MAP by 13 ± 3, 15 ± 2, and 13 ± 2 mmHg, respectively, but had no effect on HR over the 3-week treatment period. Whilst all three antihypertensive agents shifted baroreflex curves to the left, parallel to the degree of hypotension, moxonidine and rilmenidine decreased the vagal contribution to the baroreflex by decreasing the HR range of the reflex but moxonidine also increased sympathetic baroreflex range and sensitivity. By contrast clonidine had little chronic effect on the cardiac baroreflex. The present study shows that second generation agents moxonidine and rilmenidine but not first generation agent clonidine chronically shift the balance of baroreflex control of HR toward greater sympathetic and lesser vagal influences. These changes if translated to hypertensive subjects, may not be particularly helpful in view of the already reduced vagal contribution in hypertension.

## Introduction

Much evidence now supports the view that neural control of the circulation via the sympathetic nervous system is heightened early in the development of hypertension which may well contribute to severity of the disease (Esler et al., 1981; Julius and Weder, 1989). Overall sympathetic activity is not generally altered but is elevated to specific organs such as the kidney and heart in young patients with borderline hypertension (Esler et al., 1988). Furthermore, it is now well established that in chronically hypertensive man and animals there are diminished baroreceptor reflexes (Andresen et al., 1980; Mancia et al., 1986; Korner, 1989). Baroreflex control of heart rate (HR) and vasoconstrictor tone act to keep blood pressure (BP) close to a particular set point in the short term with the rapid resetting of arterial baroreceptor afferents toward any sustained new level of pressure, ensuring the reflex effectively buffers moment to moment changes in BP (Guyton, 1991). The diminished baroreflex control of HR in hypertensive subjects is of concern since recent studies have shown that this is an independent risk factor for acute myocardial infarction (Farrell et al., 1991; Fei et al., 1994). Given the increased sympathetic nervous activity to the kidney and heart and diminished baroreflex control in hypertension, there is a great deal of merit in antihypertensive treatments which not only reduce BP by reducing sympathetic outflow, but restore the baroreflex deficit.

Centrally acting antihypertensive agents, first introduced for clinical use in the early 1960's, reduce arterial pressure by decreasing sympathetic vasomotor activity (Kobinger, 1978) and have been shown to increase the sensitivity of the vagal baroreceptor-HR reflex in animals (Badoer et al., 1983). Two of the first drugs to be introduced in this category were α-methyldopa (a phenylethylamine) and clonidine (an imidazoline). Although, their mechanism of action was originally thought to involve α2-adrenoceptors, in1984, Bousquet et al. suggested that the antihypertensive effect of clonidine was more related to its imidazoline structure and suggested an action at a distinct class of receptor with affinity for imidazoline compounds, hence named the “imidazoline receptor” (Bousquet et al., 1984). Despite its effective antihypertensive action, clonidine also induces a high frequency of untoward side effects, including sedation, dry mouth, headache, depression and orthostatic hypotension (Ollivier and Christen, 1994). These effects are thought to be due to the activation of α2-adrenoceptors (Schmitt, 1977; Tibirica et al., 1989) and have limited the therapeutic potential of clonidine. Subsequently, substances with greater selectivity for imidazoline receptors were sought for use as antihypertensive agents, to reduce BP by reducing the sympathetic overactivity seen in hypertension but also to reduce side effects.

Second generation centrally acting antihypertensive agents, such as rilmenidine (an oxazoline, related structurally to imidazoline) and moxonidine (an imidazoline), were developed and found to possess a 30–60-fold higher selectivity for imidazoline sites over α2-adrenoceptors (Ernsberger et al., 1992). As such, these agents are more tolerable than clonidine, causing fewer side effects (Ollivier and Christen, 1994) with the absence of rebound hypertension upon withdrawal (Sannajust et al., 1989; Ollivier and Christen, 1994). They both induce falls in BP by activating imidazoline receptors in the rostral ventrolateral medulla to reduce sympathetic outflow, whilst rilmenidine inhibits the cardiac sympathetic baroreflex response and moxonidine inhibits the vagal component of the reflex (Head et al., 1997). This effectiveness in lowering BP combined with their longer half-lives makes them much more useful therapeutically than clonidine (Plänitz, 1984; Fillastre et al., 1988; Ostermann et al., 1988). Surprisingly, evidence for effects these centrally acting agents have on baroreflex curves is based almost entirely on acute administration of moxonidine (Khokhlova et al., 2001; Ma et al., 2007; Turcani, 2008), rilmenidine and clonidine rather than chronic administration, which is perhaps more relevant to long term therapy. There is one early study in conscious dogs using chronic but bolus dosing with rilmenidine (Spiers et al., 1990). Thus, the present study was designed to investigate and compare the effects of moxonidine, rilmenidine and clonidine on the baroreflex control of HR during chronic subcutaneous treatment over several weeks.

## Methods

### Animals

A total of 21 normotensive rabbits of either sex, derived from a multi-colored English stock maintained at the Baker IDI Heart and Diabetes Institute, each with body weight between 2.4 and 2.9 kg were included in the study. Experiments were performed in accordance with the Australian Code of Practice for the Care and Use of Animals for Scientific Purposes (1990) and were approved by the Animal Experimental Committee of the Baker Institute/Alfred Medical Research and Education Precinct.

### Chronic antihypertensive treatment

Rabbits were chronically infused with subcutaneous moxonidine (1 mg/kg/day, *n* = 6), rilmenidine (5 mg/kg/day, *n* = 8), clonidine (1 mg/kg/day, *n* = 7) or saline vehicle (*n* = 7) for 3 weeks, at a constant rate of 5 μl/h. These doses were effective chronically as determined previously (Parkin et al., 2003). Moxonidine infusions were directly preceded by an initial 280 μg/kg intravenous bolus due to its slow onset of action. Infusions were achieved using osmotic minipumps (Model 2ML2, Alza Corporation, Palo Alto, U.S.A.) implanted between the shoulder blades under local anesthetic (1% lignocaine HCL, Citanest, Astra Pharmaceuticals, North Ryde, New South Wales, Australia). Each minipump lasted 2 weeks, at which point the pumps were replaced to achieve a 3-week infusion regime. Of the 21 rabbits, 14 received one treatment whilst 7 entered a second treatment period, with a different agent, after a minimum wash-out period of 2 weeks. Three other rabbits did not complete the study for reasons unrelated to the treatments, such as minipump failure and were not included in the study results.

### Cardiovascular measurements

On each experimental day, the rabbit was placed in a wooden box designed to hold one rabbit. Minor preparatory procedures were performed under local anesthesia with 1% lignocaine HCL (Citanest; Astra Pharmaceuticals). The marginal ear vein was cannulated with a 24-gauge, 19 mm Teflon catheter (Insyte; Deseret Medical, Sansy, Utah, USA) for injections of vasoactive drugs and methscopolamine, as appropriate, and the central ear artery was cannulated with a 22-gauge, 25 mm Teflon catheter (Insyte) for measuring BP. The catheter was connected to a Statham P23Dc pressure transducer (Gould Inc., Bernie, Maryland, USA) for continuous measurements of arterial pressure and HR. We allowed a 1 h period for the rabbits to recover from the minor procedures before commencing the experiment, to permit stabilization of cardiovascular parameters. The pulsatile arterial pressure was dampened to derive the mean arterial pressure (MAP) whereas the HR was measured using a HR meter triggered from the arterial pulse. The MAP and HR signals were digitized on-line using a National Instruments data acquisition card (PC plus, Austin, Texas, USA) and a data acquisition program designed using LabVIEW graphical programming language (National Instruments, Austin, Texas, USA). Software developed at the Baker IDI Heart and Diabetes Institute was used to average and store the data over 2 s periods for subsequent analysis.

### Experimental protocols

For each drug treatment period, rabbits were studied on three separate occasions, day 0 (before treatment) and again at day 7 and day 21 (post treatment). Before commencement of each experiment, body weights were noted to ensure good health. On the first experimental day, Week 0, a pre-treatment baroreflex curve was performed, after basal MAP and HR were recorded for 20–30 min to yield control values. Following this, a minipump containing either vehicle (saline). Moxonidine, rilmenidine or clonidine was inserted between the shoulder blades for chronic subcutaneous administration. At day 7 (referred to as week 1, for convenience) and day 21 (week 3) of treatment, after resting MAP and HR were recorded for 20–30 min, the baroreflex curves were again determined.

The before- and after-treatment MAP-HR baroreflex relationships were assessed using the ramp method (Dorward et al., 1985) over the full range of physiologically relevant pressures. This method consisted of slow ramp falls and rises in MAP by intravenous infusions of phenylephrine hydrochloride (0.5 mg/ml) and sodium nitroprusside (1.0 mg/ml), respectively and was done in duplicate. Infusions took 1–2 min and the rate of change of MAP was kept between 1 and 2 mmHg per second. The sympathetic component of the reflex was then assessed during vagal blockade with intravenous methscopolamine (63 μg/kg bolus + 2.5 μg/kg/min infusion; 6 ml/h). After a second 20 min control period, measuring MAP and HR, the sympathetic baroreflex was assessed using the steady-state technique (Korner et al., 1972). Multiple step increases and decreases in MAP were reached and maintained constant for 20–30 s to allow the HR to reach a steady-state response, induced by changes in cardiac sympathetic activity. After generating the intact and sympathetic baroreflex curves, the vagal component of the reflex was determined by mathematically subtracting the sympathetic curve from the intact curve as described previously (Godwin et al., 1998). At week 2 of treatment, cardiovascular parameters were measured to ensure that the antihypertensive agents were still exhibiting an effect. Minipumps were also changed on this day.

### Analysis of MAP-HR curves

MAP and HR values were binned into 2 s intervals and fitted to a non-symmetrical sigmoid logistic function using a non-linear least squares regression program based on the Marquardt-Levenberg algorithm (Marquardt, 1963). The equation fitted was

$$\begin{array}{l} y^{\prime }=P\text{1}+\frac{P\text{2}}{\text{1}+f_{x}\cdot e^{P\text{3}\left(P\text{4}-x^{\prime }\right)}+\left(\text{1}-f_{x}\right)\cdot e^{P\text{5}\left(P\text{4}-x^{\prime }\right)}} \end{array}$$

where:

$$\begin{array}{l} f_{x}=\frac{1}{1+e^{-{\bar{c}}_{f}}\left(P\text{4}-x^{\prime }\right)} \end{array}$$

defines the function for smooth transition (between 0 and 1) which is centered about the BP_50_. The average curvature of f is given by

$$\begin{array}{l} {\bar{c}}_{f}=\frac{2\cdot P\text{3}\cdot P\text{5}}{\left|P\text{3}+P\text{5}\right|} \end{array}$$

where *P1* is the lower plateau (LP, calculated minimum HR), *P2* is the difference between the lower plateau and the upper plateau (calculated maximum HR) and *P5* and *P3* are curvature parameters indicating the rate of change as the function transitions to the plateau. *P4* is the set point MAP at half the HR range (BP_50_). The average gain of the curve (G) which is the slope between the two inflection points of the curve is G = −P2 × (*P3* + *P5*)/9.12. This logistic curve fitting routine was not forced through the resting value thus allowing it to lie away from the line of the baroreflex curve.

The contribution of the vagus was calculated as the difference between the initial curve (vagus + sympathetic) and the methscopolamine curve (sympathetic), calculated for each animal (Head, 1994). Using conscious rats, we have shown that the vagal and sympathetic components sum to produce a curve very close to the real intact vagal and sympathetic curve (Head and McCarty, 1987).

### Vagal and sympathetic contribution to basal HR

The vagal and sympathetic contributions to the maintenance of basal HR were calculated before treatment began and at weeks 1 and 3 of treatment for each experimental group, according to the methods of Head and Adams (Head and Adams, 1992). Sympathetic HR was calculated as the HR after vagal blockade (with an intravenous methscopolamine infusion) minus intrinsic HR. The latter was estimated, according to the literature, as 200 b/min for rabbits at that body weight (Zola et al., 1988; Opthof, 2000; Such et al., 2002). Vagal HR was calculated as the HR after vagal blockade (sympathetic intact) minus basal HR (vagal and sympathetic intact).

### Data analysis

Values are expressed as mean ± standard error of the mean (SEM). Treatment effects were assessed by a multi-factor repeated measure analysis of variance (ANOVA). The main effects were time (week 0, 1 and 3), drug (*n* = 4) and animals (*n* = 21). The total residual sum of squares was calculated by subtracting the between animals, between times sums of squares from the total sum of squares. This residual was then used to calculate the average treatment SEM, which indicates variation within animals. The main effect of each drug was determined from the between times by orthogonal contrasts comparing the average of week 1 and 3 vs. week 0 (control) and using the total residual sums of squares (see above). Thus, a more robust combined residual over all groups was used. For intact curves, the duplicates were averaged in the analysis. An additional analysis was performed comparing the effects (delta) of each treatment, averaged over the 3 weeks compared to vehicle. *P* < 0.05 was considered statistically significant.

### Drugs

The compounds used included moxonidine [4-chloro-word5-(2-imidazolin-2-ylamino)-6-methoxy-2-methylpyrimidine] hydrochloride (Solvay Pharma, Germany), rilmenidine [2-(dicyclopropylmethyl)-amino-2-oxazoline] dihydrogenophosphate (I.R.I., Servier, France), clonidine [2-(2,6-worddichlorophenylamino)-imidazoline] hydrochloride (Boehringer Ingelheim, U.S.A.), phenylephrine [3-hydroxy-α-((methylamino) methyl) benzenemethanol] hydro-chloride (Sigma Chemicals, U.S.A.), sodium nitroprusside [nitrosylpentacyanoferrate (III)] (Fluka Chemicals, Switzerland), scopolamine methyl [7-(3-hydroxy-1-oxo-2-phenylpropoxy)-9,9-dimethyl-3-oxa-9-azoniatricyclononane] bromide (Sigma), normal saline (Delta West, Bentley, WA, Australia), hydrochloric acid (Ajax Chemicals, Australia) and sodium hydroxide (Ajax). All drug doses are expressed as salts. Drugs used for chronic treatment were dissolved in normal saline. Moxonidine required the addition of 1M hydrochloric acid and 1M sodium hydroxide to dissolve the drug and neutralize the solution, respectively.

## Results

### Chronic effects of moxonidine, rilmenidine, and clonidine on MAP, HR, and BW

The average MAP, HR, and body weight (BW), averaged over the 4 experimental groups prior to any treatment, were 79 ± 2 mmHg, 170 ± 6 beats/min and 2.7 ± 0.09 kg, respectively. There was no difference in MAP, HR, or BW between the groups although the baseline HR in the moxonidine group tended to be slightly lower than the other groups. Subcutaneous administration of 1 mg/kg/day moxonidine and clonidine and 5 mg/kg/day rilmenidine reduced MAP by 13 ± 3, 13 ± 2, and 15 ± 2 mmHg, respectively, at week 1 of treatment [*F*_(1, 25–35)_ > 21 for each group, *P* < 0.05]. This hypotension was maintained in all 3 treatment groups throughout the 3-week infusion period. Subcutaneous infusions of saline (vehicle) had no effect on MAP (*P* > 0.5). Antihypertensive treatment [*F*(_1, 25–35)_ < 4, *P* > 0.1] and vehicle [*F*(_1, 30)_ = 0.8, *P* = 0.4] had no effect on HR and rabbits remained in good condition throughout treatment, with no change in BW.

### Chronic effects of moxonidine, rilmenidine, and clonidine on intact MAP-HR baroreflex curves

**Figure 1:**
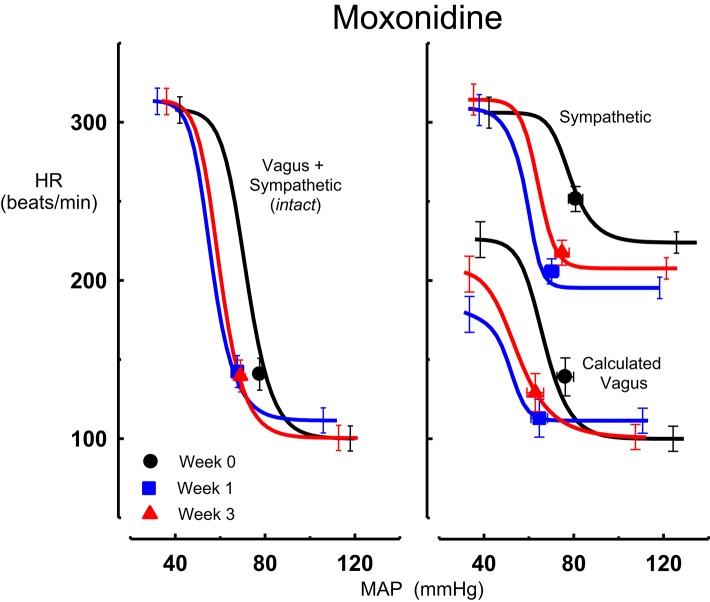
**Average sigmoid curves indicating the relationship between mean arterial pressure (MAP, mmHg) and heart rate (HR, beats/min) for the intact baroreflex curves (left panel) and the sympathetic and calculated vagal baroreflex curves (right panel) in six conscious rabbits before (weeks 0, black line, black circles on all panels) and at weeks 1 (blue line, blue square on all panels) and 3 (red line, red triangle on all panels) of moxonidine treatment**. Symbols on curves represent resting values. Error bars are average SEM indicating variation between animals calculated from the ANOVA.

**Figure 2:**
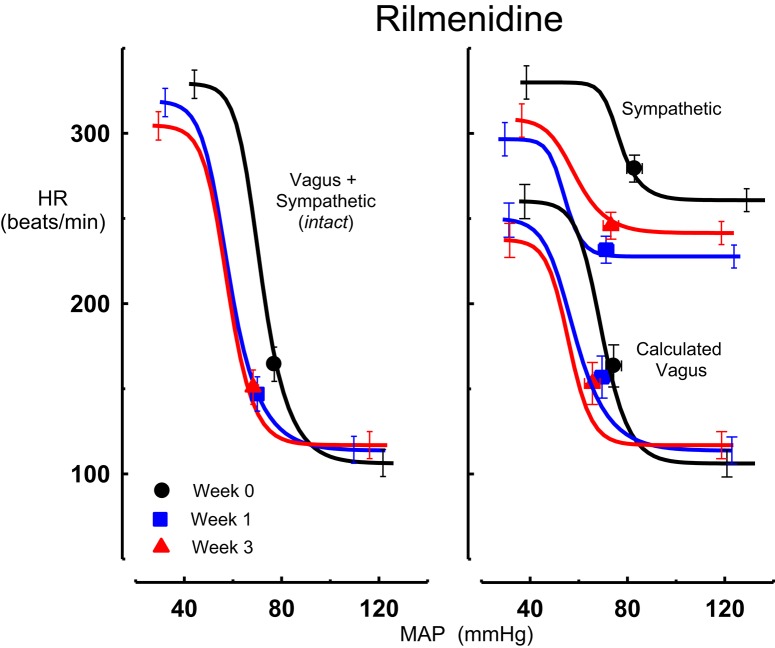
**Average sigmoid curves indicating the relationship between mean arterial pressure (MAP, mmHg) and heart rate (HR, beats/min) for the intact baroreflex curves (left panel) and the sympathetic and calculated vagal baroreflex curves (right panel) in eight conscious rabbits before (weeks 0, black line, black circles on all panels) and at weeks 1 (blue line, blue square on all panels) and 3 (red line, red triangle on all panels) of rilmenidine treatment**. Symbols on curves represent resting values. Error bars are average SEM indicating variation between animals calculated from the ANOVA.

**Figure 3:**
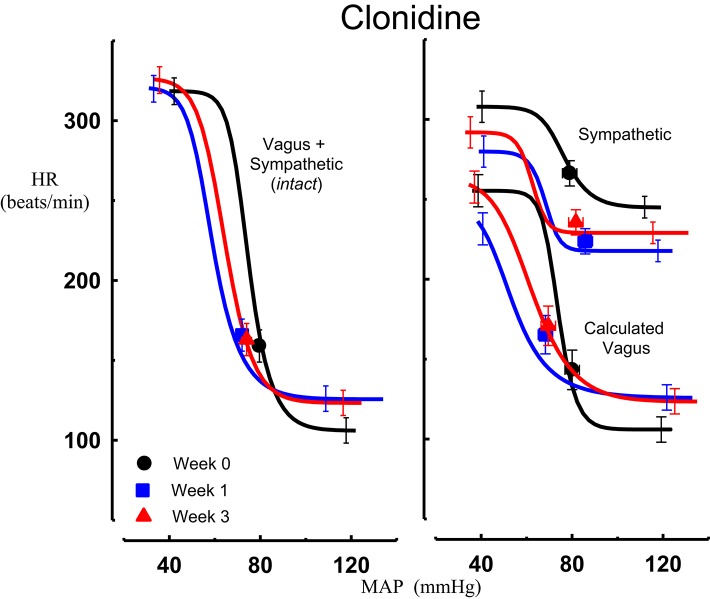
**Average sigmoid curves indicating the relationship between mean arterial pressure (MAP, mmHg) and heart rate (HR, beats/min) for the intact baroreflex curves (left panel) and the sympathetic and calculated vagal baroreflex curves (right panel) in seven conscious rabbits before (weeks 0, black line, black circles on all panels) and at weeks 1 (blue line, blue square on all panels) and 3 (red line, red triangle on all panels) of clonidine treatment**. Symbols on curves represent resting values. Error bars are average SEM indicating variation between animals calculated from the ANOVA.

All three centrally acting antihypertensive agents shifted the MAP-HR reflex curves to the left in parallel to the hypotension (Figures 1–3, left panel), resulting in decreases in average treatment BP_50_ values (−14 ± 4, −13 ± 3, and −13 ± 5 mmHg for moxonidine, rilmenidine and clonidine, respectively, *P* < 0.001) compared to pretreatment baroreflex curves (Tables 1–3). Rilmenidine reduced the intact baroreflex HR range by an average of 27 ± 9 beats/min, between weeks 1 and 3 of treatment, from pre-treatment values [*F*_(1, 64)_control vs. drug = 8, *P* = 0.007] (Figure 2; left panel; Table 2). This effect was due to a reduction in the upper HR plateau (*P* = 0.04) with the lower plateau remaining unaltered. Clonidine reduced intact baroreflex gain by an average of 26 ± 7 from week 1 of treatment (Table 3) but so did vehicle treatment (−23 ± 7%, Table 4). No other changes were seen in the baroreflex parameters for these two groups, whilst moxonidine was the only treatment that had no effect on any parameters of the intact baroreflex curve (Table 1). A comparison between treatments showed that all treatments decreased BP_50_ but that only rilmenidine reduced the HR range compared to vehicle (Table 5).

**Table 1:** **Average basal values and baroreflex parameters describing intact, sympathetic and calculated vagal MAP-HR curves before and at weeks 1 and 3 of subcutaneous administration of moxonidine (*n* = 6)**.

	**Intact**			**Sympathetic**			**Calculated vagal**		
	**Week 0**	**Week 1**	**Week 3**	**Week 0**	**Week 1**	**Week 3**	**Week 0**	**Week 1**	**Week 3**
**BASAL PARAMETERS**									
MAP mmHg	78 ± 3	68 ± 1^**^	69 ± 1^**^	81 ± 4	70 ± 1^*^	75 ± 1^*^	76 ± 4	65 ± 2^**^	63 ± 2^**^
HR b/min	141 ± 6	142 ± 12	140 ± 7	251 ± 7	206 ± 9^***^	218 ± 5^***^	139 ± 14	137 ± 9	129 ± 12
**BAROREFLEX PARAMETERS**									
Lower plateau, b/min	100 ± 7	111 ± 10	100 ± 9	225 ± 12	195 ± 5^*^	208 ± 3^*^	100 ± 7	111 ± 10	100 ± 9
Upper plateau, b/min	308 ± 5	314 ± 8	314 ± 5	306 ± 7	309 ± 11	314 ± 5	226 ± 11	200 ± 11	222 ± 20
Range, b/min	208 ± 9	202 ± 8	214 ± 9	82 ± 12	114 ± 8^*^	107 ± 6^*^	126 ± 13	89 ± 14^*^	107 ± 8^*^
BP_50_, mmHg	72 ± 4	56 ± 2^***^	60 ± 1^***^	78 ± 4	59 ± 3^**^	64 ± 1^**^	67 ± 6	49 ± 5^**^	55 ± 2^**^
Average gain, b/min/mmHg	−8.0 ± 1.2	−9.0 ± 1.2	−9.1 ± 1.3	−3.2 ± 0.7	−6.5 ± 1.1^*^	−6.2 ± 1.5^*^	−4.8 ± 1.0	−2.5 ± 1.4	−2.9 ± 1.0
Goodness of fit	96 ± 1	95 ± 1	96 ± 1	96 ± 1	96 ± 3	96 ± 1			

*Values are mean ± between animal SEM*.

* P < 0.05,

** P < 0.01 and

*** *P < 0.001 for the comparison between pre- and post-treatment*.

*MAP, mean arterial pressure; HR, heart rate; BP_50_, median blood pressure*.

**Table 2:** **Average basal values and baroreflex parameters describing intact, sympathetic and calculated vagal MAP-HR curves before and at weeks 1 and 3 of subcutaneous administration of rilmenidine (*n* = 8)**.

	**Intact**			**Sympathetic**			**Calculated vagal**		
	**Week 0**	**Week 1**	**Week 3**	**Week 0**	**Week 1**	**Week 3**	**Week 0**	**Week 1**	**Week 3**
**BASAL PARAMETERS**									
MAP mmHg	77 ± 3	70 ± 4^**^	68 ± 3^**^	83 ± 1	71 ± 3^**^	73 ± 4^**^	75 ± 5	70 ± 5^*^	65 ± 4^*^
HR b/min	164 ± 10	147 ± 8	151 ± 10	279 ± 10	232 ± 8^***^	246 ± 10^***^	163 ± 12	156 ± 7	153 ± 11
**BAROREFLEX PARAMETERS**									
Lower plateau, b/min	106 ± 6	114 ± 8	117 ± 10	261 ± 8	228 ± 11^**^	241 ± 12^**^	106 ± 6	114 ± 8	117 ± 10
Upper plateau, b/min	329 ± 9	315 ± 14^*^	305 ± 21^*^	330 ± 9	297 ± 13^*^	309 ± 11^*^	260 ± 9	250 ± 14	237 ± 17
Range, b/min	223 ± 10	205 ± 13^**^	188 ± 17^**^	69 ± 5	69 ± 13	67 ± 14	154 ± 9	136 ± 13^*^	121 ± 9^*^
BP_50_, mmHg	72 ± 4	58 ± 3^***^	58 ± 3^***^	77 ± 3	54 ± 3^***^	59 ± 5^***^	70 ± 5	58 ± 5^*^	56 ± 4^*^
Average gain, b/min/mmHg	−8.9 ± 1.1	−7.6 ± 0.9	−7.7 ± 1.2	−3.4 ± 0.7	−4.0 ± 0.7	−2.5 ± 0.7	−5.4 ± 1.1	−3.5 ± 0.8	−5.3 ± 1.2
%Goodness of fit	97 ± 1	98 ± 1	97 ± 1	95 ± 2	95 ± 2	87 ± 4			

*Values are mean ± between animal SEM*.

* P < 0.05,

** P < 0.01 and

*** *P < 0.001 for the comparison between pre- and post-treatment*.

*MAP, mean arterial pressure; HR, heart rate; BP_50_, median blood pressure*.

**Table 3:** **Average basal values and baroreflex parameters describing intact, sympathetic and calculated vagal MAP-HR curves before and at weeks 1 and 3 of subcutaneous administration of clonidine (*n* = 7)**.

	**Intact**			**Sympathetic**			**Calculated vagal**		
	**Week 0**	**Week 1**	**Week 3**	**Week 0**	**Week 1**	**Week 3**	**Week 0**	**Week 1**	**Week 3**
**BASAL PARAMETERS**									
MAP mmHg	80 ± 2	72 ± 3^*^	74 ± 3^*^	79 ± 1	79 ± 6	82 ± 3	80 ± 3	69 ± 3^**^	70 ± 4^**^
HR b/min	159 ± 8	166 ± 17	163 ± 11	266 ± 12	224 ± 12^***^	236 ± 15^***^	143 ± 10	170 ± 20	171 ± 14
**BAROREFLEX PARAMETERS**									
Lower plateau, b/min	106 ± 8	126 ± 12	123 ± 10	245 ± 10	218 ± 11^*^	229 ± 9^*^	106 ± 8	126 ± 12	123 ± 10
Upper plateau, b/min	319 ± 8	321 ± 11	327 ± 6	308 ± 8	280 ± 9	292 ± 17	255 ± 7	259 ± 10	264 ± 9
Range, b/min	213 ± 6	195 ± 10	203 ± 6	63 ± 5	62 ± 11	63 ± 10	149 ± 8	133 ± 8	141 ± 12
BP_50_, mmHg	75 ± 2	59 ± 4^***^	64 ± 5^***^	77 ± 2	62 ± 7^**^	62 ± 5^**^	74 ± 2	55 ± 5^**^	62 ± 6^**^
Average gain, b/min/mmHg	−9.7 ± 0.8	−7.1 ± 1.0^*^	−7.3 ± 0.7^*^	−2.2 ± 0.4	−4.9 ± 2.0	−4.3 ± 0.8	−7.5 ± 1.0	−2.3 ± 1.8^***^	−3.0 ± 0.6^***^
%Goodness of fit	98 ± 1	97 ± 1	98 ± 1	97 ± 1	93 ± 3	96 ± 2			

*Values are mean ± between animal SEM*.

* P < 0.05,

** P < 0.01 and

*** *P < 0.001 for the comparison between pre- and post-treatment*.

*MAP, mean arterial pressure; HR, heart rate; BP_50_, median blood pressure*.

**Table 4:** **Average basal values and baroreflex parameters describing intact, sympathetic and calculated vagal MAP-HR curves before and at weeks 1 and 3 of subcutaneous administration of vehicle (*n* = 7)**.

	**Intact**			**Sympathetic**			**Calculated vagal**		
	**Week 0**	**Week 1**	**Week 3**	**Week 0**	**Week 1**	**Week 3**	**Week 0**	**Week 1**	**Week 3**
**BASAL PARAMETERS**									
MAP mmHg	78 ± 3	79 ± 4	77 ± 2	79 ± 4	74 ± 3	79 ± 4	77 ± 4	83 ± 6	76 ± 3
HR b/min	161 ± 10	180 ± 14	162 ± 8	278 ± 12	263 ± 9	271 ± 11	149 ± 7	160 ± 14	161 ± 9
**BAROREFLEX PARAMETERS**									
Lower plateau, b/min	108 ± 9	108 ± 17	112 ± 8	251 ± 13	243 ± 11	242 ± 11	108 ± 9	108 ± 17	112 ± 8
Upper plateau, b/min	330 ± 9	324 ± 12	320 ± 9	332 ± 10	324 ± 11	328 ± 10	260 ± 15	244 ± 14	234 ± 12
Range, b/min	224 ± 14	216 ± 15	208 ± 14	80 ± 9	81 ± 6	86 ± 6	143 ± 20	136 ± 16	122 ± 15
BP_50_, mmHg	75 ± 3	74 ± 3	74 ± 3	79 ± 6	73 ± 4	79 ± 4	73 ± 5	76 ± 4	71 ± 4
Average gain, b/min/mmHg	−10.9 ± 0.8	−9.1 ± 1.4^*^	−7.8 ± 0.6^*^	−2.9 ± 0.7	−4.2 ± 1.4	−3.5 ± 1.4	−8.0 ± 1.3	−4.6 ± 1.8^**^	−4.1 ± 1.6^**^
Goodness of fit	98 ± 1	98 ± 1	97 ± 1	95 ± 1	96 ± 1	96 ± 1			

*Values are mean ± between animal SEM*.

* P < 0.05 and

** *P < 0.01 for the comparison between pre- and post-treatment*.

*MAP, mean arterial pressure; HR, heart rate; BP_50_, median blood pressure*.

**Table 5:** **Comparison of chronic effect of moxonidine, rilmenidine and clonidine on intact (vagal and sympathetic) baroreflex curve parameters compared to vehicle**.

**Basal Parameters**	**Δ Vehicle**	**Δ Moxonidine**	**Δ Rilmenidine**	**Δ Clonidine**
MAP mmHg	0.08 ± 1.57	−9.27 ± 1.24^***^	−7.85 ± 1.86^***^	−6.62 ± 1.25^**^
HR b/min	9.83 ± 6.64	0.28 ± 4.45	−15.47 ± 6.92^*^	5.4 ± 8.56
**BAROREFLEX PARAMETERS**				
Lower plateau, b/min	2.56 ± 6.01	5.99 ± 3.11	9.18 ± 5.81	18.54 ± 5.72
Upper plateau, b/min	−11.54 ± 7.14	5.5 ± 4.74	−16.16 ± 6.32	5.32 ± 5.3
Range, b/min	−8.97 ± 5.68	−0.44 ± 5.28	−26.58 ± 6.01^*^	−13.22 ± 5.97
BP_50_, mmHg	−0.52 ± 1.39	−13.64 ± 1.94^***^	−13.43 ± 2.56^***^	−13.15 ± 2.19^***^
Average gain, b/min/mmHg	2.47 ± 0.7	−1.02 ± 0.7^***^	1.14 ± 0.49	2.54 ± 0.6

*Values are mean ± between animal SEM*.

* P < 0.05,

** P < 0.01,

*** *P < 0.001 for the comparison the effect of vehicle and the other drugs (averaged over the 3 weeks)*.

*MAP, mean arterial pressure; HR, heart rate; BP_50_, median blood pressure*.

### Chronic effects of moxonidine, rilmenidine, and clonidine on the sympathetic component of the MAP-HR baroreflex curves

**Figure 4:**
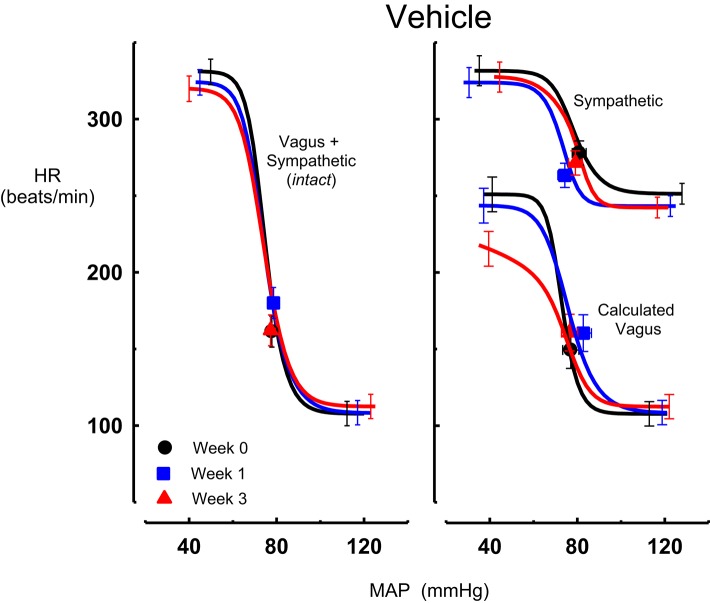
**Average sigmoid curves indicating the relationship between mean arterial pressure (MAP, mmHg) and heart rate (HR, beats/min) for the intact baroreflex curves (left panel) and the sympathetic and calculated vagal baroreflex curves (right panel) in seven conscious rabbits before (weeks 0, black line, black circles on all panels) and at weeks 1 (blue line, blue square on all panels) and 3 (red line, red triangle on all panels) of vehicle treatment**. Symbols on curves represent resting values Error bars are average SEM indicating variation between animals calculated from the ANOVA.

Intravenous methscopolamine, by blocking cardiac vagal activity, increased HR in all four experimental groups, at weeks 0, 1, and 3 of treatment (*P* < 0.01). Assessment of the remaining sympathetic component of the cardiac baroreflex at these times showed that antihypertensive treatment with moxonidine, rilmenidine and clonidine caused the baroreflex curves, at weeks 1 and 3, to be shifted to the left of the pre-treatment sympathetic curve for each group (Figures 1–3; right panels). This leftward shift reflects the hypotension produced by the agents and thus, the BP_50_ values for these curves were correspondingly lower than at week 0 (Tables 1–3). The major effect of vagal blockade on the baroreflex was to reduce the HR range by two thirds (66%) of the intact curve, principally by an increase in the level of the lower HR plateau. Moxonidine, rilmenidine, and clonidine all caused decreases in the lower plateau of the sympathetic baroreflex curves, from week 1 of treatment (*P* < 0.01), compared with their respective pre-treatment curves (Figures 1–3; right panels; Tables 1–3). With rilmenidine treatment, this decrease in the lower plateau was accompanied by a similar decrease in the upper plateau, averaging 27 ± 10 beats/min, between weeks 1 and 3 of treatment, resulting in a parallel downward shift of the sympathetic curves with no change in HR range (P control vs. drug = 1). This was probably due to a baroreflex independent inhibition of cardiac sympathetic activity. In contrast, this decrease in lower plateau was not accompanied by a similar decrease in the upper plateau with moxonidine (P control vs. drug = 1) and clonidine treatment (P control vs. drug = 0.06), with the upper plateau remaining unaltered, resulting in an increase in the sympathetic HR range in moxonidine treated animals by an average of 34 ± 10% (*P* = 0.01), but no change in HR range with clonidine. Moxonidine also caused a 98 ± 41% (*P* = 0.04) increase in the sympathetic baroreflex gain, compared with pre-treatment values, due mostly to the increase in HR range. Vehicle treatment did not affect any sympathetic baroreflex parameters, with the curves at weeks 1 and 3 being superimposed on the pre-treatment curve (Figure 4; right panel).

A comparison between treatments on the sympathetic curves showed that all treatments decreased BP_50_ but there were no other effects of baroreflex parameters compared to vehicle (Table 6).

**Table 6:** **Comparison of chronic effect of moxonidine, rilmenidine and clonidine on sympathetic baroreflex curve parameters compared to vehicle**.

**Basal Parameters**	**Δ Vehicle**	**Δ Moxonidine**	**Δ Rilmenidine**	**Δ Clonidine**
MAP mmHg	1.95±3.44	−8.36±2.56^*^	−10.69±1.9^**^	1.58±3.53
HR b/min	−11.5±5.1	−39.8±7.2^*^	−40.6±8.7^*^	−36.7±10.6^*^
**BAROREFLEX PARAMETERS**				
Lower plateau, b/min	−11.1±6	−22.5±7.3	−26.2±6.6	−23.7±8.3
Upper plateau, b/min	−7.3±9.4	5.6±8.4	−27.3±7.3	−22±11.7
Range, b/min	3.8±7.8	28.1±10.2	−1.1±7.5	1.7±6.7
BP_50_, mmHg	4.01±4.51	−16.94±2.63^**^	−20.46±3.06^***^	−13.9±5.03^**^
Average gain, b/min/mmHg	−1.2±0.85	−3.13±0.93	0.12±0.73	−2.36±1.1

*Values are mean ± between animal SEM*.

* P < 0.05,

** P < 0.01,

*** *P < 0.001 for the comparison the effect of vehicle and the other drugs (averaged over the 3 weeks)*.

*MAP, mean arterial pressure; HR, heart rate; BP_50_, median blood pressure*.

### Chronic effects of moxonidine, rilmenidine, and clonidine on the vagal component of the MAP-HR curves (calculated)

The calculated vagal curves as shown in Figures 1–4, right panels, and the parameters, indicated in Tables 1–4, were calculated by subtracting the sympathetic sigmoid curve from the original intact curve and therefore represent approximately 66% of the normal baroreflex HR range. Moxonidine and rilmenidine both caused a decrease in the calculated vagal HR range, similarly at weeks 1 and 3 [*F*_(1, 64)_ control vs. drug = 4, *P* = 0.04, *F*_(1, 64)_ control vs. drug = 5, *P* = 0.04, respectively], averaging 29 ± 13 and 25 ± 12 beats/min. However, this decrease in range was not due to any significant changes in either the upper or lower plateaus alone. As seen in the intact curve, clonidine and vehicle treatment similarly reduced the gain of the calculated vagal baroreflex [*F*_(1, 64)_ control vs. drug = 15, *P* = 0.0003, *F*_(1, 64)_ control vs. drug = 8, *P* = 0.005, respectively], with average changes of 65 ± 16 and 46 ± 21%, respectively, after 1 week of treatment (Tables 3, 4). As there were no changes in gain in the sympathetic curve, we have confirmed that the reductions in gain seen in the intact curves with clonidine and vehicle treatment were due to a reduction in the gain of the vagal component of the baroreflex.

### Contribution of vagus and sympathetic to HR

**Figure 5:**
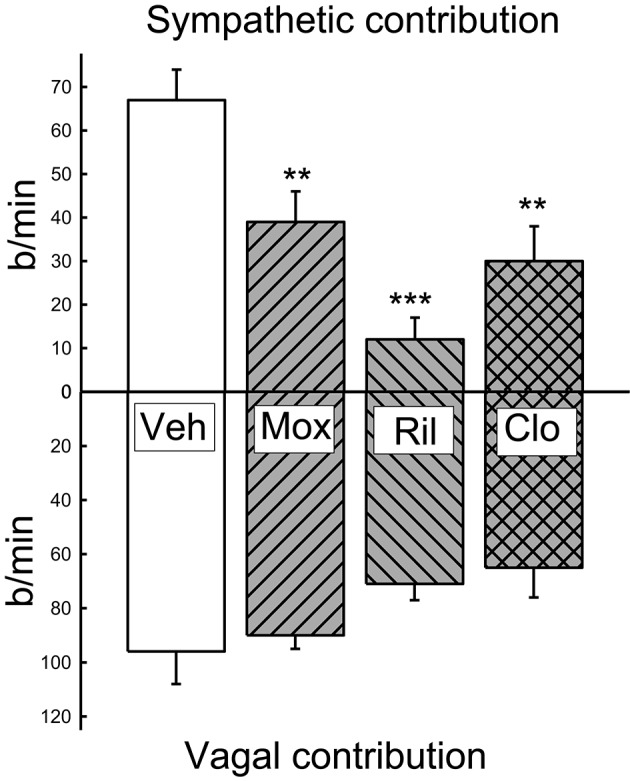
**Average contribution of the sympathetic and vagal influences on resting heart rate at 1 and 3 weeks of treatment in vehicle (Veh), moxonidine (Mox), Rilmenidine (Ril) and clonidine (Clo)**. Vagal contribution calculated as the change in heart rate induced by the administration of methscopolamine. Sympathetic component calculated as the heart rate under methscopolamine minus the intrinsic heart rate. The latter was estimated as 200 b/min according to the literature for rabbits at that body weight (Opthof, 2000). ^**^P < 0.01 and ^***^P < 0.001 for the effect of treatment compared to vehicle.

At the end of the 3-week treatment period, the contribution of the cardiac vagus to resting HR was maintained in all treatment groups although there was a tendency for a small reduction in the moxonidine and clonidine treated rabbits (−40 ± 10 b/min and −42 ± 10 b/min respectively) compared to baseline. However, this was not different to the change observed in the vehicle group (−21 ± 13 b/min). Nor was there any difference between the absolute values between groups (Figure 5). The contribution of the cardiac sympathetic to resting HR was reduced by all 3 treatments (Figure 5).

## Discussion

The present study examined the chronic effects of moxonidine, rilmenidine and clonidine on the vagal and sympathetic components of the cardiac baroreflex in conscious rabbits. We found that all drugs shifted curves to the left in line with the hypotension which is an expected finding related to baroreceptor resetting. This is a well-known phenomenon and does not usually involve a change in central baroreflex function (Dorward et al., 1982). However, despite the similar degree of hypotension produced by all three agents, there were unexpectedly quite disparate effects of the drugs on cardiac baroreflexes. Rilmenidine and moxonidine reduced HR range of the vagal curves while clonidine and vehicle treatment had little effect. For rilmenidine, this was also observed in the intact curves (vagal and sympathetic combined) but not for moxonidine. Moxonidine increased the range of the sympathetic curves while rilmenidine, clonidine and vehicle treatments showed no change in this parameter over 3 weeks. By contrast, all 3 agents reduced the lower plateau of the sympathetic baroreflex and also the sympathetic component of the resting HR. Thus, the resting value moved from the middle of the curve to lie much closer to the lower plateau. There was also a tendency for a lesser vagal contribution to resting HR which to some degree offset the reduced sympathetic resulting in no long term change in basal HR in treated rabbits. Thus, moxonidine, and to a lesser extent rilmenidine, increased the proportion of the sympathetic component in the baroreflex, whilst decreasing the vagal component. Not only was the range of the reflex affected but the gain of the sympathetic curve was doubled by moxonidine over the 3-week treatment period. A similar trend was observed with clonidine but it did not reach the significance value (*P* = 0.06). Thus, second generation agents rilmenidine and moxonidine attenuated the vagal component of the curve while moxonidine produced a marked facilitation of the cardiac sympathetic curve. Importantly, these results suggest that chronic effects on baroreflexes cannot be assumed by the class of agent in relation to centrally acting antihypertensive drugs and need to be individually assessed.

Rilmenidine and to a lesser extent clonidine treated animals exhibited a downward shift of the cardiac sympathetic reflex curve at weeks 1 and 3 of treatment without any effect on the sympathetic reflex range. Hence these changes are due to baroreflex independent alterations. Moxonidine reduced the lower plateau like rilmenidine but the upper plateau was not altered. Thus, there was an increase in the sympathetic HR range. Moxonidine also increased the gain of the sympathetic baroreflex curve, suggesting that with moxonidine treatment, cardiac sympathetic activity is more sensitive to changes in pressure. Thus, with much reduced basal sympathetic activity after moxonidine, the capacity to increase HR with a hypotensive challenge is increased. The effects of moxonidine on the baroreflex, as determined in this chronic study, suggest that moxonidine should have a more prominent role in hypertensive therapy especially since acute studies have reported a beneficial inhibition of the cardiac sympathetic particularly in relation to treating the sympathetic overactivity related to hypertension (Head and Malpas, 1997). These cardiac sympatho-inhibitory properties also applied to rilmenidine and clonidine except that the sympathetic range was maintained and not increased. Also in all cases the contribution of the sympathetic to the resting HR was reduced. The difference between moxonidine and rilmenidine and clonidine is that the latter two drugs will produce a lesser increase in cardiac sympathetic activation in the presence of hypotension. The clinical implications of the sympathetic modulation effect of moxonidine if translated to humans may have contributed to the issues of higher mortality in heart failure patients observed in the MOXCON trial (Cohn et al., 2003).

We have previously assessed the acute effects of these agents on vagal and sympathetic components of the baroreflex in conscious rabbits (Godwin et al., 1998). A comparison of the published acute and the current chronic effects has been made in Table 7. Surprisingly absolutely none of the acute effects of these agents has been observed during chronic treatment. Acutely, rilmenidine and clonidine increased the vagal range of the reflex which is the opposite in the case of rilmenidine to the chronic effects. Rilmenidine also acutely reduced the range of the sympathetic curve but had no effect chronically. Moxonidine on the other hand acutely reduced the gain of the intact baroreflex curves but had no effect chronically. This was due to a chronic increase in the gain and range of the sympathetic component and a reduction in the range of the vagal component. None of these effects of moxonidine was observed acutely. Clonidine acutely increased the vagal component of the baroreflex curves which has been suggested to be due to activation of α2-adrenoceptors but had no effect chronically. The explanation for this may be due to a down regulation in contribution of central α2-adrenoceptors which have been shown to reduce in number with as little as 6 days treatment (Hamilton et al., 1992). Our own study using antagonists at imidazoline and α2-adrenoceptors during chronic administration of moxonidine, clonidine and rilmenidine found no selectivity for imidazoline receptors over α2-adrenoceptors for HR effects suggesting they were largely mediated through the latter receptor (Parkin et al., 2003). This contrasts the hypotension which for moxonidine, rilmenidine, and clonidine is largely mediated through imidazoline receptors (Parkin et al., 2003). Interestingly, the antagonists after chronic treatment caused a rebound tachycardia in rilmenidine and clonidine groups but no effect in moxonidine treated rabbits, suggesting that there was actually a greater tonic inhibitory influence of α2-adrenoceptors (Parkin et al., 2003). Given the inhibition of the sympathetic component particularly by rilmenidine (see Figure 5), this suggests that the cardiac sympatho-inhibition is mediated by activation of α2-adrenoceptors.

**Table 7:** **Comparison of chronic and acute effect of moxonidine, rilmenidine and clonidine on baroreflex curve parameters**.

	**Moxonidine**		**Rilmenidine**		**Clonidine**	
	**Acute**	**Chronic**	**Acute**	**Chronic**	**Acute**	**Chronic**
BP_50_, mmHg	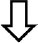	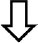	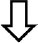	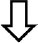	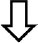	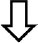
Basal HR	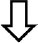	–	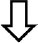	–	–	–
Range V+S	–	–	–	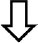	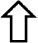	–
Range V	–	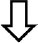	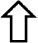	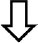	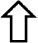	–
Range S	–	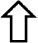	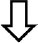	–	–	–
Average gain V+S,	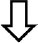	–	–	–	–	–
Average gain V,	–	–	–	–	–	–
Average gain S,	–	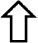	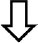	–	–	–

*Up arrows indicate an increase in each parameter compared to baseline either acutely (data from Godwin et al., 1998) or as an average at 1 and 3 weeks post chronic treatment. Down arrows indicate a decrease in each parameter. V+S indicates intact curve with both vagal and sympathetic components. V indicates the calculated vagal contribution and S indicates the sympathetic contribution after blockade with methscopolamine*.

The disparate effects of acute and chronic administration are a salient reminder of the value in performing studies which more closely reflect the long term treatments usually associated with hypertension. Surprisingly there have been very few studies examining baroreflex changes with chronic treatment with any of the centrally acting agents. A study in conscious dogs using 15 days of a single daily bolus dose of rilmenidine reported no change in the cardiac baroreflex with chronic treatment but an increase in gain with acute treatment (Spiers et al., 1990). By contrast we did not observe any change in gain either acutely (Godwin et al., 1998) or chronically (current study) in conscious rabbits. A 4 week treatment of 8 mildly hypertensive patients demonstrated a small increase in baroreflex sensitivity after 4 weeks of rilmenidine but this study was an open label trial with no randomisation, crossover or placebo thus making interpretation difficult (Finta et al., 2006).

The main strengths of our study are the use of a within animal experimental design, use of conscious animals, assessment of intact and sympathetic components of the baroreflex curve, use of full sigmoidal curves rather than straight line regression and the inclusion of a vehicle time control. The limitations are that we used only one dose for each drug but these were based on dose response curves performed with chronic treatment in our previous studies (Parkin et al., 2003). All drugs produced a similar degree of hypotension which enabled us to compare them across all the baroreflex variables. A further limitation is that we estimated the vagal contribution by subtracting the sympathetic component from the intact curve rather than determining it directly. However, this technique has been used successfully previously (Godwin et al., 1998).

In conclusion, our study shows that chronic treatment with first and second generation antihypertensive agents produces small but important modulation of the baroreflex control of HR. The main effects were to shift the curves leftward and inhibit basal cardiac sympathetic activity. Second generation agents moxonidine and rilmenidine but not first generation agent clonidine chronically shifted the balance of baroreflex control of HR toward greater sympathetic and lesser vagal influences. These changes if translated to hypertensive subjects, may not be particularly helpful in view of the already reduced vagal and greater sympathetic activity in hypertension. First generation agent clonidine had virtually no chronic effect on cardiac baroreflexes in the present study.

## Author contributions

MP, KL, SB, and GH: Contributed to the analysis of the data and writing of the manuscript.

### Conflict of interest statement

The authors declare that the research was conducted in the absence of any commercial or financial relationships that could be construed as a potential conflict of interest.
